# A Conceptual Approach for the Design of New Catalysts for Ammonia Synthesis: A Metal—Support Interactions Review

**DOI:** 10.3390/nano13222914

**Published:** 2023-11-08

**Authors:** Javier Arroyo-Caire, Manuel Antonio Diaz-Perez, Mayra Anabel Lara-Angulo, Juan Carlos Serrano-Ruiz

**Affiliations:** Materials and Sustainability Group, Department of Engineering, Universidad Loyola Andalucía, Avda. de las Universidades s/n, Dos Hermanas, 41704 Seville, Spain; jarroyo@uloyola.es (J.A.-C.); madiaz@uloyola.es (M.A.D.-P.); malangulo@uloyola.es (M.A.L.-A.)

**Keywords:** green ammonia, mild conditions, electron transfer, 3rd-generation catalysts

## Abstract

The growing interest in green ammonia production has spurred the development of new catalysts with the potential to carry out the Haber–Bosch process under mild pressure and temperature conditions. While there is a wide experimental background on new catalysts involving transition metals, supports and additives, the fundamentals behind ammonia synthesis performance on these catalysts remained partially unsolved. Here, we review the most important works developed to date and analyze the traditional catalysts for ammonia synthesis, as well as the influence of the electron transfer properties of the so-called 3rd-generation catalysts. Finally, the importance of metal–support interactions is highlighted as an effective pathway for the design of new materials with potential to carry out ammonia synthesis at low temperatures and pressures.

## 1. Introduction

Ammonia is one of the most widely consumed chemicals worldwide. The high annual ammonia production (ca. 190 MMt·year^−1^ in 2020 [1]), along with the growing demand for nitrogen-containing fertilizers [2], represents an environmental challenge since the synthesis of this commodity is currently carried out by the conventional Haber–Bosch process, one of the most energy-consuming industrial processes (ca. 200–400 bar of pressure and 400–600 °C of temperature), responsible for approximately 1.2% of global anthropogenic CO_2_ emissions [3]. 

In addition, like most industrial processes nowadays, the hydrogen used for ammonia synthesis (see Equation (1)) is mainly obtained from non-renewable sources (e.g., natural gas reforming), which eventually results in a considerable environmental impact.

(1) $$N_{2}+3H_{2}\to 2NH_{3}$$

In order to make this industrial system economically feasible, the Haber–Bosch process is carried out in large central facilities which, in reality, contain two large industrial plants in one (a steam methane reforming plant and an ammonia synthesis plant). Considering the growing concern around Greenhouse Gas (GHG) emissions, there is a pressing need to increase the sustainability of the process by coupling ammonia synthesis with renewable hydrogen production via wind- or photovoltaic (PV)-driven water electrolysis. However, effective coupling of both technologies requires ammonia synthesis to be carried out under significantly milder pressure and temperature conditions than those used in the Haber–Bosch process. The use of lower temperatures and pressures in the synthesis reactor paves the way for designing smaller non-centralized ammonia plants, which could act as local fertilizer producers [4]. This green ammonia concept represents a shift from traditional high-scale centralized production to low-scale distributed generation [3].

Apart from its main use as a raw material for fertilizers (approximately 80% of the total ammonia produced [3]), ammonia has recently demonstrated a potential use as a hydrogen carrier in virtue of its high hydrogen content (ca. 18 wt%) [1,3,5,6]. Moreover, ammonia is much easier and also cheaper to transport than hydrogen, based on its low vapor pressure (≃10 bar). Actually, ammonia has a well-established international supply chain and could become a suitable and effective pathway to transport hydrogen. Thus, ammonia could be considered as a potential and attractive carbon-neutral feedstock for the near future energy applications.

Based on the rising importance of ammonia production under mild conditions, the feasibility of new methods such as chemical looping [7,8,9], photocatalysis [10,11], photothermal catalysis [12], plasma [13,14], electrochemical-assisted synthesis [15,16] and other methods at the embryonic stage of development, such as mechanocatalytic synthesis [17], is being analyzed. However, although important advances, all these technologies are still at the very early stage of investigation and additional studies are required to demonstrate their efficiency and scalability in realistic industrial conditions [5,18].

Regarding these promising technologies, they have some advantages but also many challenges to compete effectively with more mature thermocatalytic ammonia synthesis [18]: plasma synthesis can be carried out under mild conditions and can afford transients typical from renewables. However, ammonia synthesis rates are still low, and it is a high energy-consuming process. Electrochemical synthesis also enables operating under mild conditions and reduces CO_2_ emissions by the use of H_2_O as a hydrogen source, but it has some drawbacks, such as the competition of both nitrogen and hydrogen to be adsorbed on the TM and low ammonia selectivity. In addition, photocatalytic synthesis also enables ammonia synthesis under mild conditions, but dinitrogen promotion and activation performance targets are not good enough yet. Therefore, it seems that thermocatalytic ammonia synthesis is the most advantageous and developed technology to date, with a high future potential, since there are many research possibilities to improve the current technology.

On the other hand, the design of novel materials for ammonia thermocatalytic synthesis has experimented important progress in recent years, especially since the elucidation of the positive synergy between the metal functionality (usually ruthenium) and a wide variety of promoters and supports. In recent decades, different materials have been investigated with the aim to optimize the design of suitable catalysts [5,18,19].

Investigation of these materials is motivated by the potential environmental advantages of improving the ammonia synthesis activity of current industrial catalysts by several orders of magnitude, which relies on a reduction in energy consumption in the current Haber–Bosch ammonia plants. As aforementioned, decreasing temperature and pressure can potentially result in a decrease in CO_2_ emissions since less energy is required to obtain ammonia. Additionally, the possibility of coupling ammonia synthesis carried out by novel catalysts with renewable energies would increase the environmental attractiveness of the process even more, since the necessary hydrogen could be obtained from water electrolysis, making ammonia synthesis a carbon-neutral process [5].

Based on the recent and fast development of new strategies for the design of new materials with high activity for ammonia synthesis under mild conditions and on the complexity of the phenomenology associated with each kind of catalyst, this review provides: (i) a general view of the progress made through the different generations of catalysts, (ii) a comprehensive explanation of the different mechanisms involved in the thermocatalytic ammonia synthesis reaction and (iii) the future outlook and perspectives for the development of new materials. Unlike several works published to date [18,20,21,22,23,24,25], the aim of this review is to unveil the fundamentals of electron transfer and metal–support interactions, since these concepts are key aspects for the development of the so-called 3rd-generation catalysts for ammonia synthesis. In fact, since the discovery of the C12A7:e^−^ electride, investigations into metal–support interactions led to a drastic change in the paradigm of catalyst development [26]. As demonstrated in this review, the promotion of the electron transfer between the support and TM can be used as a reasonable criterion for the design of efficient ammonia synthesis materials.

## 2. Thermocatalytic Ammonia Synthesis

### 2.1. Historical Evolution of Catalysts for Ammonia Synthesis

The Haber–Bosch process is the predominant route for ammonia production worldwide [3]. The ammonia synthesis reaction (Equation (1)) is limited by the high stability of the N_2_ molecule (N≡N bond energy of 945 kJ·mol^−1^), which is one of the strongest known chemical bonds and requires precise forces to work at high temperatures (400–600 °C) to increase the reaction rate. Since this reaction is exothermic (−92 kJ·mol^−1^), it is necessary to operate at high pressures (200–400 bar) to overcome the thermodynamic limitations derived from working at those temperatures [27]. The industrial process has been operating since the early 20th century on the so-called 1st-generation catalysts based on iron oxides [21]. Industrially, the most commonly used catalysts are those derived from magnetite (Fe_3_O_4_). However, iron oxides by themselves do not exhibit significant activity so these materials are generally supported on Al_2_O_3_ and promoted by other components such as K_2_O, CaO or MgO [20,21,28,29]. The ammonia synthesis reaction over Fe is sensitive to the structure rather than to electronic interactions, with Fe(111) being the most active facet [30]. Although 1st-generation promoted iron-based catalysts are widely used (mostly due to their low price), they present low intrinsic activities, thereby being forced to operate at high temperatures to achieve industrially acceptable activities. Operation at high temperatures harms equilibrium conversion, thereby needing to operate at elevated pressures and increasing the complexity of the industrial process. The classical Haber–Bosch process is therefore non-compatible with renewable hydrogen production processes (e.g., water electrolysis), which normally operate under milder operation conditions.

At the end of the 20th century, a new class of catalysts based on ruthenium over carbon were developed in an attempt to reduce synthesis pressures and temperatures [20,21]. The discovery and optimization of these 2nd-generation catalysts (i.e., ruthenium over carbon normally promoted with Cs or Ba) led to a new industrial process for the production of ammonia called the Kellogg Advanced Ammonia Process (KAAP), in which it is possible to obtain yields of 40–50%, at lower temperatures (370–400 °C) and pressures (50–100 bar) as compared to the conventional Haber–Bosch process using iron oxides as the catalyst [22]. 

Moreover, Ru-based catalysts have been demonstrated to be highly effective towards ammonia synthesis, and more active than the conventional Fe-based catalysts [31]. However, ruthenium is a low abundant noble metal which hinders its industrial use due to its high cost. Therefore, since the release and development of the KAAP process, several strategies for the design of new catalyst based on Ru have been evaluated in order to increase the activity and stability of these kind of catalysts, with the objective of minimizing the amount of noble metal in the catalyst.

As in the case of iron-based catalysts, generally, the dissociative adsorption of N_2_ is the Rate Determining Step (RDS) on ruthenium catalysts, as it will be explained in more detail in the Section 2.3. The high activity of Ru-based catalysts for ammonia synthesis, in comparison with other transition metals (from now, TMs), is normally associated with the active B5 site (i.e., a configuration of three ruthenium atoms in a layer with two further ruthenium atoms in the layer just above), which works as an active center for N_2_ cleavage [22,31]. The concentration of B5 sites on the catalysts is dependent on their particle size, so several studies have been focused on the rational design of nanomaterials (i.e., reducing the size of the particles to a nano scale) in order to promote the amount of B5 sites on the catalysts. It was demonstrated that catalysts with a size ranging from 1.8 to 2.5 nm exhibit the highest amount of this specific kind of active sites, so different synthesis procedures have been checked to decrease the size of the particles, which is generally higher [32]. Furthermore, from a structural point of view, as it is common in heterogeneous catalysis, a high specific surface and a proper porosity is desirable, and this is also generally obtained by decreasing the particle size. 

However, even considering a reduction on the size of the particles and the presence of a noble metal, a sole Ru over carbon catalyst commonly exhibits poor ammonia synthesis activity [33] and ruthenium-based materials are thus commonly promoted by the addition of some extra compounds: alkali and alkaline earth promoters have been demonstrated to be very effective at achieving high activities towards ammonia production [22]. Among them, cesium and barium are the most widely used, although their beneficial effects are attributed to different phenomena. As an example, the addition of different amounts of cesium to a ruthenium over ceria (Ru/CeO_2_) catalyst, as well as the impact of the synthesis route, were recently explored [34]. It was concluded that both the concentration of the cesium promoter and the procedure followed for the preparation of the catalyst could influence the performance of the material for the ammonia synthesis reaction. From these results, it was observed that the optimal behavior in terms of ammonia production and Turnover Frequencies (TOF) was obtained by adding the cesium to the support before ruthenium impregnation, with an optimal load of Ru of 2% wt. and a Cs/Ce rate of 0.35. It was suggested that larger amounts of promoter could block the Ru active sites, decreasing the activity as a result. The beneficial effect encountered once the correct concentration of cesium was used was attributed to the electron-donating effect of cesium as well as an improvement on the resistance of the so-made catalyst to hydrogen poisoning that commonly affect the ruthenium-based catalysts (see Section 2.3 for more details). The interaction of cesium and the support can change the electronic properties of the catalyst, increasing its performance due to the synergetic effect of ruthenium and cesium. The electronic promotion encountered with the addition of cesium was also previously reported by several authors and it is well accepted that the low electro negativity of alkali and alkaline materials promotes the mentioned beneficial electronic effect [33].

Similarly, the addition of barium to different ruthenium-supported catalysts has been demonstrated to significantly increase the activity for ammonia production [33,35,36,37]. The presence of barium on the catalyst seems to also influence the electronic properties of the material in some extent due principally to the presence of Ba^0^ [38]. However, it was suggested that barium promotion comes from changes in the catalyst structure, by increasing the highly active B5 sites [22].

It is interesting to note that despite the enhancement obtained by the addition of cesium being much higher than that encountered when barium acts as a promoter [33], it has been demonstrated that cesium degrades more rapidly than barium on a ruthenium supported over yttria-stabilized zirconia catalyst [35], thus increasing the industrial attractiveness of the latter. Therefore, it is clear that the activity is not the only effect to be considered for a successful scalability of thermocatalytic ammonia synthesis technology.

Based on the different effects observed with Cs or Ba (i.e., electronic and structural, respectively), the synergy between these two promoters was also investigated on a ruthenium over carbon catalyst [39]. It was claimed that a Ru catalyst promoted by Cs and Ba exhibited higher activities than those promoted by a sole species (Cs or Ba). This is pretty interesting since it demonstrates that for a successful design of a catalyst for ammonia synthesis under mild conditions, every single beneficial effect should be considered to maximize its activity.

The promotion effect of other alkali elements, like potassium and sodium, has also been analyzed [38,40], although their beneficial effect over ruthenium catalysts is lower than that obtained with cesium or barium. In fact, it is well accepted that the effectiveness of the promoters increases inversely with the electronegativity of the element used [41]. Nonetheless, despite the beneficial effect observed with K or Na, it is not as high as that obtained with Cs or Ba, the addition of both Na and K to Ru-based catalysts results in a remarkable promotion of ammonia synthesis [38,40] and it is worth to consider these elements as promising promoters since they are much more abundant on earth than cesium and barium, which could facilitate their spread into the industrial scale. It was recently demonstrated [42] that ammonia synthesis activities are higher when potassium is added to Ru/MgO and Ru/CeO_2_ catalysts. Thus, an enhancement of more than double when 4% wt. of K is added to the Ru/CeO_2_ catalyst was reported, and a threefold increase for K-Ru/MgO catalyst as compared to the unpromoted Ru/CeO_2_ and Ru/MgO.

It is also interesting to note that the role of promoters is intimately associated with the nature of the support and the effect can vary significantly [22]. For instance, although the addition of small quantities of Cs (as low as a relation of Cs/Ru = 1) to a Ru/MgO catalyst could significantly increase the activity, a relation much higher (Cs/Ru = 10) did not result in a substantial enhancement when Al_2_O_3_ is used as support. In the case of alumina acting as support, it is suggested that the promoter interacts with the acid sites of the Al_2_O_3_, not covering the Ru surface, which results in a “deactivation” of the beneficial effect of the Cs [22].

Nowadays, a Ru-based catalyst promoted by Cs which uses high surface graphite carbon (HSGC) as support is commonly used at the industrial scale in the KAAP process [22]. However, at the same time, a wide range of materials are still being investigated intensively as potential supports, seeking a high activity and stability towards ammonia synthesis under mild conditions. Since the development of the KAAP process, 2nd-generation Ru-based catalysts have been further researched, with a special emphasis on the support morphology and its properties [22].

In this context, some common oxides, like MgO, ZrO_2_, CeO_2_, and Al_2_O_3_, which are among the most commonly used heterogeneous catalyst supports, have been proposed as a possible way to enhance ammonia synthesis activity and stability. Among them, it has been demonstrated that those oxides with a certain degree of basicity can help to increase the catalytic performance for ammonia synthesis [34,43]. In fact, as aforementioned, some authors claim that acid sites (as those in Al_2_O_3_) can strongly interact with ammonia once produced, limiting its desorption and finally decreasing the overall activity as a result [22], although they could promote the activation of the dinitrogen. The importance of the basicity on the ammonia synthesis activity was also highlighted by Miyahara et al. [43] using Ru-based catalysts supported on lanthanoids oxides. 

Authors encountered a positive relation between the basicity of the catalyst and the TOF, demonstrating that the ammonia production rate increases when light lanthanoid oxides (e.g., Ru/Pr_2_O_3_, Ru/CeO_2_, Ru/LaO_3_) were used as supports, which means that the enhancement on the ammonia synthesis activity was intimately related with the catalysts with the highest basic site concentration.

On the other hand, the effect of oxygen vacancies of the support has also been identified as an important parameter to consider for the ammonia synthesis activity [44,45]. It has been demonstrated by several works that a high amount of oxygen vacancies can promote the nitrogen adsorption and its activation in photochemical applications [46,47,48]. Based on this idea, the effect of oxygen vacancies was studied, analyzing ceria-based supports with different morphologies for a thermocatalytic ammonia synthesis [44]. The higher activity of CeO_2_ nanorods over the CeO_2_ nanocubes under the same operating conditions (400 °C, 1 MPa) was attributed to the presence of a high concentration of oxygen vacancies, along with a low crystallinity (which favors the presence of Ru^4+^ ions, instead of metallic Ru particles).

Moreover, the importance of the oxygen vacancies for the ammonia synthesis reaction has been further investigated [45]. The effect of doping a Ru over CeO_2_ catalyst with samarium (Sm) (Ru/Sm_2_O_3_-CeO_2_) was studied and it was demonstrated that the addition of Sm^3+^ (up to 7% wt.) to ceria increases the concentration of oxygen vacancies, which greatly increased the ammonia synthesis activity as compared to that catalyst with no Sm. The enhancement observed once Sm was used was related to the improvement of the reducibility properties of Ru and ceria, which could ensure a more efficient breakdown of the nitrogen triple bond by the donation of the electrons from the partially reduced ceria to the Ru metal particle, along with the increase in the hydrogen desorption capacity, which can prevent hydrogen poisoning and thus promote the nitrogen adsorption.

As previously demonstrated in several works, an enhancement into the electronic properties of the catalyst improves the nitrogen dissociation into atoms before reacting with hydrogen to form ammonia. Inspired by this idea, researchers focused their attention into some materials with high ability of donating electrons with the aim of increasing the activity of ammonia production under mild operating conditions. Thus, inorganic electrides, ionic materials in which the electrons serve as anions [49], were considered as a promising support for the active metal.

In this context, there are several electrides that have demonstrated a good performance acting as support for the ruthenium in the ammonia synthesis reaction as compared to 2nd-generation catalysts [26,50,51,52]. Among them, it is remarkable the work developed with the electride [Ca_24_Al_28_O_64_]^4+^(e^−^)_4_ (so called C12A7:e^−^) [26,53]. The material obtained hydrothermally and latterly reduced at 1173 K (HT-C12A7:e^−^) functions as a high efficiency catalyst for ammonia synthesis at atmospheric pressure and 615 K, with activities higher than the majority of conventional catalysts used. Further works demonstrated not only the well-known beneficial effect by the donation of electrons, but also the crucial role of the hydride ion mobility (caused by the reversible hydrogen storage capacity of the C12A7:e^−^), which significantly increases the resistance to hydrogen poisoning [50,54]. In addition, and also important to note, it was demonstrated that the RDS was the formation of N-H_x_ species, since the apparent activation energy of Ru/HT-C12A7:e^−^ is lower than that corresponding to nitrogen dissociation step, in contrast to conventional catalysts.

On account of the importance of the electron mobility, a wide variety of supports have been tested during the last decade, with the objective of shifting the RDS from nitrogen dissociation towards the formation of NH_x_ species, most of them enhancing metal–support interactions via promoting electron donation between support and TM, since this strategy leads to globally better-performing materials. The majority of these supports, which constitute the basis of the 3rd-generation catalysts for ammonia synthesis, have the particular feature of permitting a continuous flow and high mobility of electrons by their capacity of location inside the structural cages of the material. Apart from the electride materials aforementioned, there are other types of supports, such as nitrides, amides and hydrides, whose performance for low-temperature NH_3_ production have been studied.

On the one hand, the presence of nitrogen vacancies in nitride-derived materials, such as CeN or LaN, activates the adsorption of both N_2_ and H_2_, promoting the catalytic performance even for metals that, in principle, exhibit a low activity towards ammonia production like Ni. Moreover, pure CeN nanoparticles are able to generate NH_3_ at 400 °C (250 μmol·g^−1^·h^−1^ and 1450 μmol·g^−1^·h^−1^ at 0.1 MPa and 0.9 MPa, respectively) which probes that presence of nitrogen vacancies (N_V_) derived from the nitride species over the catalyst surface is able to activate both the hydrogen and the nitrogen even with no TM [55,56,57], since it works as a second active center for both nitrogen dissociation and hydrogen activation. On the other hand, alkaline earth amide materials are also proposed as effective supports for Ru (also for Co) [58]. In fact, Ru over Ba-doped Ca(NH_2_)_2_ have been demonstrated to be much more active for low-temperature NH_3_ synthesis than such as benchmark catalyst as Cs-Ru/MgO (100-fold higher at 260 °C). Ru/Ba-Ca(NH_2_)_2_, which exhibits a core–shell configuration and a mesoporous structure with a large surface area, is able to suppress hydrogen poisoning and highly activate N_2_ dissociation (in this case, the rate-limiting step is the formation of N-H_x_ species rather than N_2_ cleavage) [37].

Further, the utilization of hydrides as support (details of hydride uses are discussed later in Section 2.2) has been demonstrated to be an effective strategy to enhance the formation of NH_3_ under mild conditions. These kinds of materials, which are able to function as an electron and hydrogen donors, have been demonstrated to exhibit an excellent performance for ammonia production under mild conditions. Hydrides are also very versatile since they can be used as supports for many different TMs [59], which is one of the hardest handicaps of 2nd-generation ammonia synthesis catalysts. Furthermore, the internal structure of alkaline hydrides can be modified by the addition of some elements, like fluorine, to increase the performance of the catalyst [60], which can enable conducting the ammonia synthesis reaction at temperatures as low as 100 °C.

A recent alternative to nitrides, hydrides, electrides and rare earth-based oxides can be found in the use of intermetallic compounds, whose understanding is of outstanding interest since they have promising properties in the field of catalyst design for many different applications and particularly for ammonia synthesis [24,61,62,63]. Intermetallic materials can be used indistinctly as supports for Ru or as a compact catalyst where the TM is located in the intermetallic matrix. On the one hand, when they are used as supports for Ru, intermetallics exhibit strong metal–support interactions (SMSI): work functions as low as 2.7, 3.2 and 3.2 eV were measured for LaCoSi, LaFeSi and LaMnSi, respectively, which are lower than those of pure La (3.5 eV), thus showing a surface electride-like behavior, which not only leads to a strong metal–support electron transfer, but also to an enhanced thermal stability of Ru. In addition, interactions between Ru and the TM from the support matrix (Co, Fe or Mn) also play an important role in the Ru dispersion and particle size, and in the global kinetic mechanism as well, given that the presence of a second active center is reported for these catalysts: while N_2_ dissociation takes place on Ru sites, hydrogenation of N^*^ dissociated atoms to form NH_x_ species occurs over La surfaces [64]. On the other hand, single-phase LaRuSi intermetallic material was reported as an efficient catalyst for ammonia synthesis [65], since stronger chemical bonds between Ru and La are assumed to improve the electron transfer [63], whose activity is attributed to a dual behavior as an electride material, similarly to aforementioned intermetallic supports [24,61,62,63], as well as the reversible exchange between lattice hydride ions and anionic electrons that takes place [65], leading to an activation energy as low as 40.4 kJ·mol^−1^. Further investigations concluded that (La,Ce,Pr)RuSi intermetallics show an excellent stability to air and moisture [66], with no relevant changes in ammonia synthesis rate after half a year of exposure to ambient air during the storage of the material. Moreover, a successful strategy to enhance those non-loaded intermetallic catalysts based on chemical etching was reported [66]. This process is based on the partial selective removal of the rare earth element over the surface, which leads to an increase in the number of Ru sites on the catalyst surface, as well as an increase in the surface area [66], leading to a 3-fold increase in the ammonia synthesis rate of LaRuSi after 5 h treatment with EDTA-2Na 5 mM. An additional simulation work based on first-principles calculations revealed that a key aspect to explain the high performance of LaRuSi is the presence of a bowl active site, composed by four surface La and one subsurface Si atoms, in which N_2_ dissociation is driven by electrostatic and orbital interactions [67]. Furthermore, a highly versatile electron charge for the Si atom located in the bowl Si atom ensures an efficient NH_x_ species desorption by electrostatic repulsion, where the required energy for NH_x_ species formation is assumed to be independent of the N_2_ adsorption energy, which accounts for an additional approach to circumvent the traditional relation between these two energies [68].

Most recent works on the thermocatalytic ammonia synthesis field are focused on enhancing the performance of materials by two different strategies: on the one hand, the use and understanding of rare earth-derived novel materials is on high demand, since they show different action mechanisms as ammonia synthesis supports, with a very versatile chemical nature [63,69,70]. It is to underline that catalysts based on ruthenium over lanthanum or cerium oxyhydrides [Ru/(Ce-La)H_3−2x_O_x_] prepared by solid-state reactions between pure hydride and oxide components show much superior ammonia synthesis activity and stability than their corresponding pure oxide and hydride precursors [71], with a maximum rate for x = 0.25 in case of lanthanum oxyhydrides and x = 0.75 for cerium oxyhydrides. The superior performance of these compounds is attributed to enhanced nitridation resistance over pure lanthanoid hydride species due to better hydrogen ion stabilization in the oxygen lattice, thus ensuring improved electron donation towards Ru.

On the other hand, modification and reorganizing of internal structures of catalysts combine for a rising approach for design of novel materials: first of all, regarding the ruthenium oxyhydrides aforementioned [71], very recent findings suggest that hydrogen diffusivity in the support can be unexpectedly increased by lowering the “x” value, i.e., lowering the available the number of sites for hydrogen diffusion, since knock-off mechanism takes place, which accounts for repulsive Coulombic interactions between H- ions inside the support matrix that are more significant than the generation of active vacancy sites [72]. This finding can lead to improved hydrogen activation and ammonia synthesis performance. Secondly, there are promising results involving the addition of dopant species inside the support matrix, as reported in the experimental study of CaFH solid solution as a stable support for Ru [60]. In this work, it was demonstrated that the addition of fluorine (F^−^) to CaH_2_ highly enhances the original hydride performance at low temperatures and pressures: the strategy of introducing F^−^ anions into a CaH_2_ matrix led to an increase on the electron donation of the original material. The electron repulsion between H^−^ and F^−^ and the weakening of the Ca-H bond (Ca-F ionic bond is stronger than Ca-H one) enables reducing the temperature required for the H_2_ migration, i.e., the work function of the support is reduced from 2.7 eV (CaH_2_) to 2.2 eV (CaFH). As a result, the doping with F^−^ generated unprecedently high ammonia synthesis rates, with some measurable activity even at temperatures as low as 50 °C and a pressure of 1 bar (~50 µmol g^−1^ h^−1^) and an activation energy of 20 kJ·mol^−1^ in the range 50–150 °C, which is approximately a half of that observed on other novel 3rd-generation catalysts [20]. As a third example, nano-powdered LaNi_5_ as a compact alloy showed a superior performance than any other Ni-based catalyst and even comparable to some Ru-based materials [73]. The high activity of this material is attributed to the self-reorganization of the original homogeneous LaNi_5_ matrix into a core–shell structure with a surface layer made of LaN under ammonia synthesis reaction conditions, which acts as a second active center for N_2_ dissociation, while both LaN surface layer and Ni encapsulated in the core act as hydrogen activators, leading to an ammonia synthesis rate as high as 4500 µmol g^−1^ h^−1^ at 400 °C and 0.1 MPa. Finally, a very recent experimental study of a barium-doped Co/La_2_O_3_ catalyst revealed that TOF was increased more than 12 fold, under the same experimental conditions, as the reduction temperature of the catalyst was increased from 500 to 700 °C [74]. This enhancement was ascribed to the encapsulation of Co by a nano-fraction of BaO-La_2_O_3_ over the catalyst surface, whose formation process was enabled by the low melting point of the Ba precursor Ba(OH)_2_, which could melt and migrate together with La_2_O_3_ towards the Co surface, thus creating a self-organized core–shell structure made by a core of Co and a low-crystalline layer of BaO-La_2_O_3_ containing voids, enabling reactant gases to break through it. The formation of this layer further enhances the metal–support electron transfer, leading to an improved ammonia synthesis performance.

To summarize, the evolution of the most relevant catalysts mentioned in this section is shown in the Table 1.

The ammonia synthesis reaction features are described in the Section 2.2, with a particular accent on the 3rd-generation catalysts.

### 2.2. Ammonia Synthesis Reaction Fundamentals

**Figure 1:**
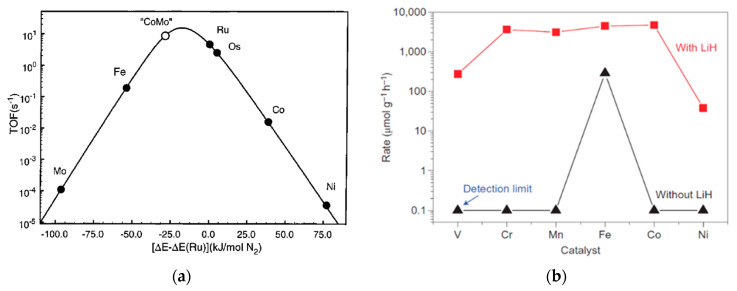
Ammonia synthesis performance for different TMs. (**a**) Volcano plot derived from microkinetic studies by the application of the Sabatier principle. Reproduced with permission [68]. Copyright 2001, American Chemical Society, and (**b**) qualitative comparison of the performance of the TMs with and without the LiH supporting effect. Reproduced with permission [59]. Copyright 2017, Springer Nature.

Theoretical investigations based on microkinetics with the application of the Sabatier principle [75] showed that there is a limit for the catalytic performance among the 3d and 4d TMs, given by the linear relation that exists between the activation energy for N_2_ dissociation and the N≡N binding energy: the so-called scaling relation, i.e., there is a fixed TOF, which measures the ammonia synthesis rate for a specific TM, given by the scaling relation [76]. These studies resulted into the volcano plot showed in the Figure 1a, which predicts Fe, Ru and CoMo bimetallics as the most suitable TMs for ammonia synthesis catalysts.

However, the discovery of the stable electride C12A7:e^−^ demonstrated that the scaling relations can be broken by the addition of a second alternative active center to the material, apart from the TM [59], which is typically associated with the support. As mentioned in the Section 2.1, from this moment, the 3rd-generation catalysts arose, showing unprecedent high catalytic activities, derived from metal–support interactions [20]. Indeed, the most remarkable finding from these 3rd-generation catalysts is the positive circumvent of the scaling relation, which was a limitation for 2nd-generation catalysts. This phenomenon is represented in the Figure 1b [59], where the ammonia synthesis rate is shown when some TMs are supported on LiH (red line) and without LiH (black line), in contrast with the volcano plots derived from the application of the Sabatier principle (Figure 1a) [68]. As can be observed, a qualitative comparison shows that some TMs traditionally unsuitable for ammonia synthesis based on their low binding energy to dissociate N_2_ can be used due to the promotional effect of the second active center originated by the presence of the support action.

As a result of the growing interest in the field of the metal–support interactions associated with the 3rd-generation catalysts, great efforts are being made by several groups in order to unravel the reaction mechanism and to understand the implication of the promotional effects.

### 2.3. Ammonia Synthesis Reaction Mechanism

The reaction mechanisms for the 3rd generation of catalysts have been analyzed [23,77]: two commonly accepted routes for ammonia synthesis have been suggested, as shown in the Table 2: the dissociative route, in which N_2_ molecule is cleaved by the electronic promotion effect, i.e., the electron transfer from the TM to the π* antibonding orbitals of N_2_; and the associative route, in which the N_2_ dissociation step is circumvented. 

In the dissociative mechanism, the electrons encaged (*) in the support (in the form of bare electrons, H^−^ or other form, depending on the support nature) are transferred to the TM, thus increasing the electron density, which leads to an enhancement of the dissociation of N_2_ (1–2). Moreover, the dissociation of H_2_ takes place on the TM surfaces and the H^*^ adatoms spill over into the support (7–8). Then, the activated N* and H* species react to form ammonia (3–6).

In the associative mechanism, in contrast, N_2_ direct dissociation is bypassed and molecular dinitrogen is directly hydrogenated by dissociated hydrogen to form ammonia (4–10).

Apart from the action of metal nitrides and other specific materials involving the associative mechanism, the dissociative route seems to be the predominant pathway for most 3rd-generation catalysts studied [21]. As aforementioned, the N_2_ activation and cleavage (1–2) is the RDS. Though operating at high temperatures and pressures was typically the way to boost ammonia synthesis, the use of electronic promoters has been the route to improve the RDS performance in terms of operating under mild conditions [20,21].

**Figure 2:**
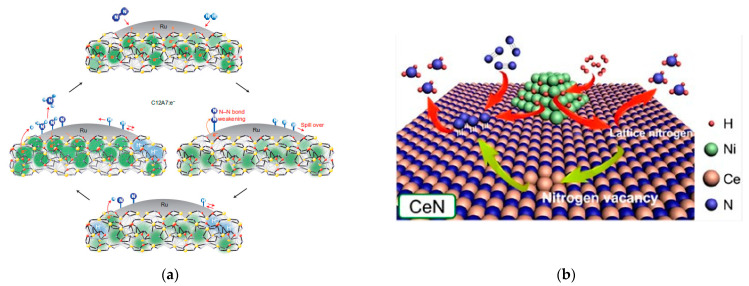

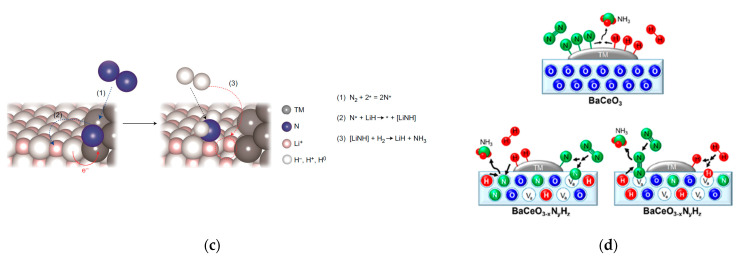
Ammonia synthesis mechanism scheme for (**a**) Ru/C12A7:e^−^ (electride). Reproduced with permission [26]. Copyright 2012, Springer Nature, (**b**) Ni/CeN (nitride). Reproduced with permission [56]. Copyright 2020, American Chemical Society, (**c**) TM-LiH (hydride). Reproduced with permission [59]. Copyright 2016, Springer Nature, and (**d**) CaCeO_3−x_N_y_H_z_ (perovskite oxy-nitride hydride). Reproduced with permission [78]. Copyright 2019, American Chemical Society.

Regarding the ammonia synthesis mechanism, high experimental effort has been put towards determining the influence of the metal–support interactions in some catalysts and their electronic promotion, including the roles of H and N layers for hydrides and nitrides, respectively. In addition to the influence of the particular N and H layers on the associative pathway, the global scheme is similar for different supports and corresponds to the dissociative mechanism. In the Figure 2, a scheme for the ammonia synthesis mechanism is shown for catalysts with different configurations: in the case of Ru/C12A7:e^−^ (electride, Figure 2a) [26] and TM/LiH (Hydride, Figure 2c) [59], the reaction is prone to follow a dissociative adsorption pathway for N_2_, whose cleavage is enhanced by the electronic promotion from the support, in the form of bare electrons (C12A7:e^−^) or H^−^ (TM/LiH), respectively. This electronic promotion is explained in detail in the Section 2.4. However, for the Ni/CeN catalyst (nitride, Figure 2b) [56], N_2_ reacts following an associative mechanism through its interaction with the N* layer of the support, which reacts with the dissociated H* to form ammonia. In addition, there are some complex materials, such as the TM/BaCeO_3-x_N_y_H_z_ (rare earth perovskites oxy-nitride hydrides, Figure 2d), which make the two possible routes feasible [78].

Significant differences can be found in the particular fundamentals for each support type, as shown in the Figure 2. However, any catalyst developed to date follows one of the catalytic mechanisms shown in the Table 2, even those which are not promoted by the action of second active centers, such as alkali/alkaline oxides or activated carbons. Oxygen vacancies, which arise as a consequence of the reversible reducibility of rare earth oxides and some particular surface electrides [63], lead to the promotion of electron transfer towards the TM, which drives nitrogen dissociation following the dissociative pathway, greatly outperforming traditional 2nd-generation catalysts kinetic mechanisms. Alkali, alkaline hydrides and their derivates perform on a similar way, since hydride ions can be exchanged by electrons which also boost the dissociative cleavage of dinitrogen. However, in this case, the transfer of electrons in the form of H^−^ anions makes hydrides attractive since they act as second active centers for hydrogen dissociation [20]. A combination of oxygen vacancies and reversible H^−^ donation makes oxyhydrides a promising branch of catalysts to consider for their high performance [71]. As aforementioned, nitrides typically follow the associative route: a reversible uptake of N* towards the nitride surface boost direct nitrogen reaction with dissociated hydrogen, which globally enables the catalyst to circumvent the nitrogen dissociation step, as demonstrated in several works [56,73]. In this case, the driving force for ammonia synthesis is directly related to the formation of NH_x_ species. According to these fundamentals, the nitride second active center for nitrogen dissociation enables the use of non-noble TMs with reasonable performances, which represents an advantage to most catalysts driven by the dissociative route, since electron transfer is not usually applicable to these materials.

It is noteworthy that novel technologies in addition to traditional thermochemical catalysis, such as photothermal or electrochemical catalysis, are ruled by either associative or dissociative mechanisms. As an example, in photothermal synthesis of ammonia using Ni/TiO_2_ as catalyst, solar light activates and dissociate nitrogen by its interaction with photoelectrons trapped into the generated oxygen vacancies, while hydrogen is dissociated by the thermocatalytic action of Ni [12]. Therefore, although the driving force is photocatalytic action, the global mechanism follows the dissociative pathway. In contrast, chemical looping enables carrying out nitrogen and hydrogen cleavage steps independently. Ammonia synthesis materials used in chemical looping, such as lithium–palladium hydrides [7], act as intermediate cyclic carriers, i.e., the complex system Li-Pd-H leads to fix dinitrogen following a dissociative route, which is stored in the form of Li_2_NH. This carrier is then hydrogenated to produce ammonia.

**Figure 3:**
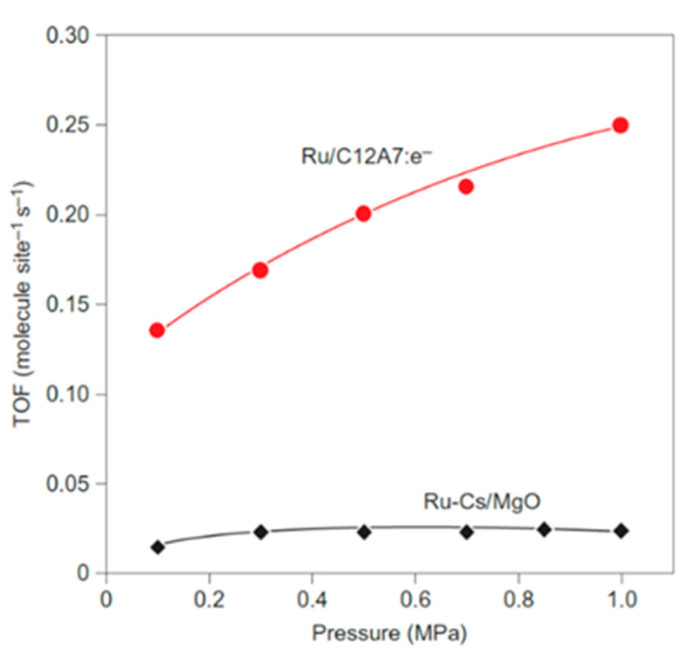
TOFs for Ru/C12A7:e^−^ and Ru-Cs/MgO at 360 °C Reproduced with permission [26]. Copyright 2012, Springer Nature.

There is another key positive effect of the metal–support electronic promotion, which is the reduction in hydrogen poisoning, typical from 2nd-generation Ru-based catalysts. In that case, the dissociative adsorption of H_2_ competes with the N_2_ adsorption and cleavage and thus the reaction orders with respect to H_2_ are usually negative, due to the accumulation of hydrogen adsorbed species inside the catalyst active sites. This phenomenon is more remarkable at higher pressures, which represents a challenge for the industrial operation of the Haber–Bosch system [5,59]. However, the electron donation from the support to the TM ensures a continuous supply of H^*^ by a reversible hydrogen migration [18,20,21,79], preventing hydrogen poisoning. Furthermore, the presence of H^−^ anions in the support from hydrides and their derivates is interesting since it gives a simultaneous backup of electrons and H^−^ (thus ensuring positive H_2_ reaction orders) [59,80,81]. The impact on the electron transfer between support and TM in terms of hydrogen poisoning is shown in the Figure 3, were ammonia synthesis performance as a function of the operating pressure from 1 to 10 bar is plotted for Ru/C12A7:e^−^ (3rd generation) and Cs-Ru/MgO (2nd generation), at 360 °C [26]. In the case of Ru-Cs/MgO (black line), there is not a significant effect of increasing the operating pressure, as long as hydrogen poisoning suppresses its thermochemical and kinetic benefits. However, the ammonia synthesis rate increases with pressure for the Ru/C12A7:e^−^ (red line), since hydrogen poisoning is circumvented.

Another relevant aspect of the 3rd-generation catalysts resides in their application, since electron donation has been demonstrated to provide a high activity for ammonia decomposition to some catalysts, such as Ru/CeO_2_ [82]. A reversible production of green ammonia has an outstanding potential, especially considering ammonia as a carbon neutral high-density fuel which can be directly used in fuel cells [83]. Furthermore, there are another environmental applications in which electron transfer plays a key role, such as CO_2_ upgrading, in which oxygen vacancies from CeO_2_ promote the thermochemical methanation process, catalyzed by Ru/CeO_2_ [84].

As a global result of the phenomenology explained up to this point, the ammonia synthesis rate of the 3rd-generation ammonia synthesis catalysts is superior by at least one order of magnitude to 2nd-generation Ru-based catalyst [20,21,79] and their activation energies much lower (40–60 kJ mol^−1^ and 80–100 kJ mol^−1^, respectively).

### 2.4. Metal—Support Interactions: Electron Transfer

The fundamentals behind the electron transfer between supports and TMs have been slightly explained and contrasted with the wide experimental background on the 3rd-generation ammonia synthesis catalysts. However, some works have recently been published in order to clear out the insights of metal–support interactions.

**Figure 4:**
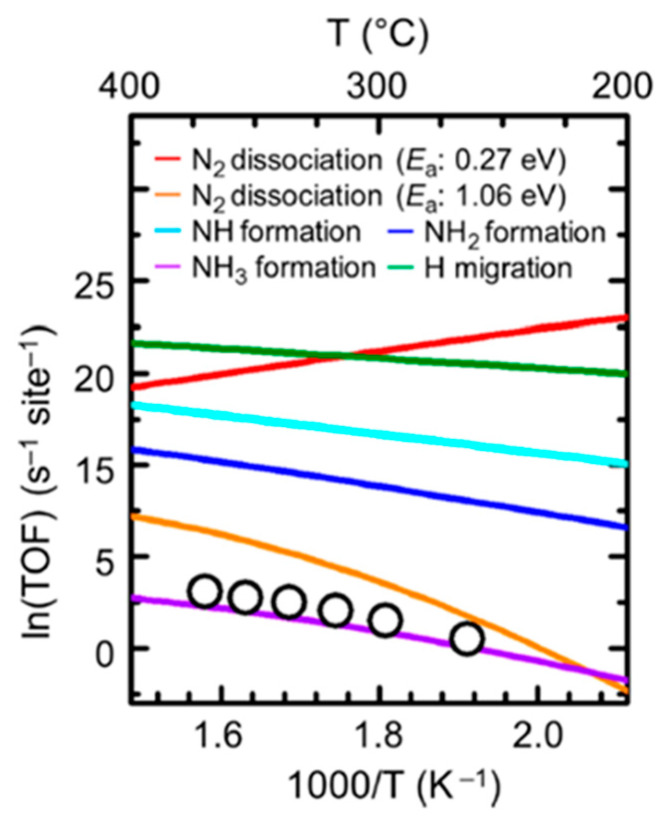
Comparison of ammonia synthesis rate for every elementary step (Table 2) and experimental results (white dots) for the Ru/Ca_2_NH catalyst at 1 bar, as a function of temperature [77].

Particularly, a theoretical analysis of ammonia synthesis was performed, based on the application of DFT and microkinetic modelling for the Ru/Ca_2_NH catalyst at atmospheric pressure and a temperature range of 300–400 °C [77]. The TOFs were simulated for every elementary step and contrasted with the experimental work performed for the same material [50], assuming the dissociative route as the correct pathway. The results shown in the Figure 4 demonstrated that the RDS is shifted from the N_2_ dissociation towards NH_x_ species formation, which is in accordance with previous experimental works [20,85].

More precisely, the RDS was demonstrated to be given by the reaction of the dissociated N_2_ and the hydrogen from the H lattice of the support in the TM–support interface through an associative mechanism, which is consistent with previous findings for other hydride catalysts [20,85].

In addition, the key aspects of the electron transfer among TM and support for electride-like catalysts, as well as the support requirements for an efficient electronic promotion have been recently pointed out [86]: the difference between the work functions (the required energy to move an electron from the Fermi level to the void, i.e., the minimum energy to extract an electron from a solid) of the TM (4.2–5.1 eV) and support (2.1–3.0 eV) generates an electron transfer from the latter to the former and active sites on the structure are created. Therefore, it seems that the higher the difference in the work functions of TM and support, the better the electronic promotion.

**Figure 5:**
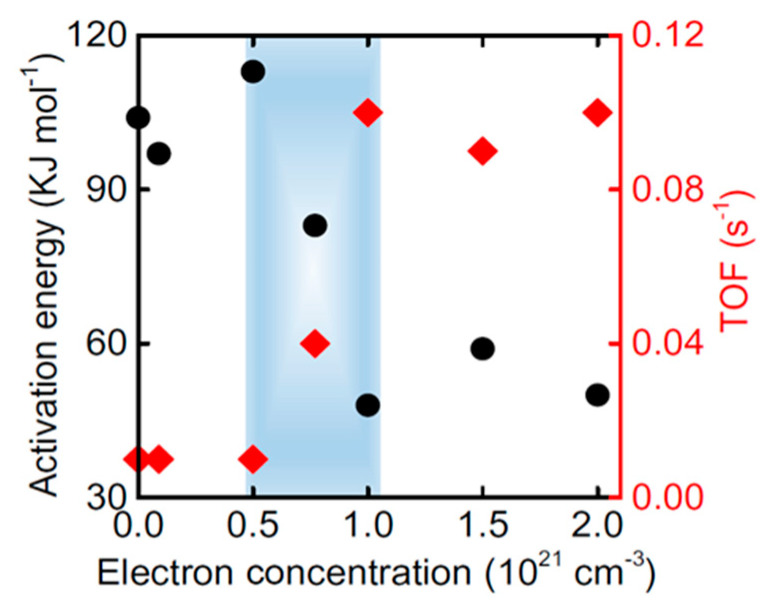
Ammonia synthesis activation energy and TOF as a function of the support electron concentration, for the Ru/C12A7:e^−^ catalyst. Reproduced with permission [86]. Copyright 2021, Springer Nature.

The activation energy for the Ru/C12A7:e^−^ catalyst, as well as ammonia synthesis performance as a function of the electron concentration in the support are shown in the Figure 5 [86]: for the original material (12CaO·7Al_2_O_3_), which corresponds to electron concentrations lower than 0.5 × 10^21^ cm^−3^, the activation energy is similar to that found for 2nd-generation catalysts. However, as the electrons are doped on the material and their concentration is increased beyond 1 × 10^21^ cm^−3^, the support acquires its electride behavior, thus leading a dramatic increase on its performance, due to the enhancement of the N_2_ dissociation, which explains the previously discussed shift on the ammonia synthesis RDS [20,77,85].

Furthermore, in the particular case of the supports based on hydrides, the deposition of the TM on the hydride support creates TM–hydride bonds, which gives an electride-like nature to some originally non-electride materials. This property, which is promoted by the formation of H^−^ at the TM–support interface, originates the so-called surface electrides [79]. Surface electrides such as Ru/Ca_2_NH and Ru/Sr_2_NH show very high ammonia yields at atmospheric pressure and temperatures below 300 °C, comparable with those of Ru/C12A7:e^−^ [87] and their activity is attributed to the above-mentioned surface electride nature.

In addition, a simple analysis of the electronic promotion of catalysts and their performance can be performed based on work function gaps between TM and support: for example, regarding the analysis developed about the performance of different 3d TMs (from the groups 6–9) supported on LiH [59], their ammonia synthesis rate is ordered as follows: Co~Fe > Cr > Mn, which matches with the order of the work functions of these TMs (Co: 5.0 eV, Fe: 4.8 eV, Cr: 4.5 eV, Mn: 4.1 eV).

In contrast, from an experimental study about the performance of CaCN_2_ as a support for Ru [88], it was concluded that the ammonia synthesis rate of CaCN_2_ is slightly higher than that of other high performance electrides such as Ca_2_N and C12A7:e^−^, even considering the higher work function of CaCN_2_ (3.95 eV) compared with Ca_2_N and C12A7:e^−^ (2.6 and 2.4 eV, respectively). In this case, the higher activity of the Ru/CaCN_2_ is attributed to a better dispersion of Ru nanoparticles on the support.

Moreover, the activities of CeH_2+x_, LaH_2+x_ and YH_2+x_ when supporting Ru are 1.63, 1.27 and 0.56 mmol g^−1^ h^−1^ at 300 °C and 1 bar, respectively [89], whose order does not correspond to the former metals work function sorting (2.9 eV for Ce, 3.5 eV for La and 3.1 eV for Y).

**Figure 6:**
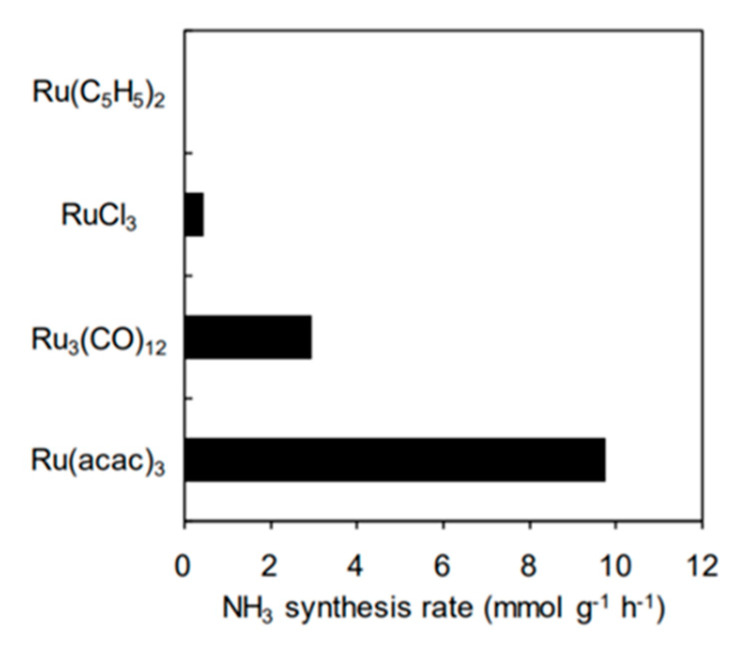
Ammonia synthesis rate for different Ru precursors supported on Ba-Ca(NH_2_)_2_, at 9 bar and 300 °C. Reproduced with permission [37]. Copyright 2018, Wiley Online Library.

In addition, there are many other aspects influencing the performance of surface electride catalysts: for example, the ammonia synthesis rate varies approximately 1 order of magnitude for different Ru precursors when supporting it on Ru/Ba-Ca(NH_2_)_2_ by impregnation of Ba and Ca with liquid NH_3_ at −40 °C [37], as shown in the Figure 6.

Apart from this, the recently synthesized Ru/CaFH solid solution has 1 order of magnitude higher performance than any other catalyst tested at atmospheric pressure [37,43], but the estimated work function of CaFH is 2.2 eV, which is similar to that of C12A7:e^−^ (2.4 eV) and even higher than that of Ca(NH_2_)_2_ (2.1 eV). Therefore, the F^−^ repulsion effect and the superior electron donation from the CaFH cannot be solely explained by a work function analysis, but considering alternative phenomenology, as reported in Section 2.1.

**Table 1:** Classification and industrial progress of catalysts for ammonia synthesis.

Classification	Catalysts Nature	Catalysts Examples	Industrial Applications ^1^	Ref
1st-generation catalysts	Iron-based (magnetite)	Fe/Al_2_O_3_	-	[21]
	Promoted iron-based	K-Fe/Al_2_O_3_-CaO	Haber–Bosch process	[20,21,28,29]
2nd-generation catalysts	Ruthenium on carbon	Ru/C	-	[22]
	Doubly promoted ruthenium on non-functional supports	(Ba-Cs)-Ru/MgO (Ba-Cs)-Ru/C	KAAP process	[22]
3rd-generation catalysts	Electrides	Ru/C12A7:e^−^, Ru/Ca2N:e^−^	-	[26,85]
	Rare earth oxides	Ru/CeO_2_, Ru/PrO, Ba-Co/La_2_O_3_		[44,74,90]
	Hydrides	Ru/LiH, Ru/Ca_2_NH, Ru/CaFH		[59,60,87]
	Nitrides	Co_3_Mo_3_N, Ni/CeN		[56,91]
	Oxyhydrides (nitrides)	Ru/CeH_3-2x_O_x_, BaCeO_3-x_N_y_H_z_		[37,71]
	Intermetallics	LaRuSi, CeRuSi, LaCoSi, LaNi_5_		[66,67,73,92]

^1.^ Relevant industrial scaling was only carried out in the Haber–Bosch (K-Fe/Al2O3-CaO) and KAAP ([Ba-Cs]-Ru/MgO, [Ba-Cs]-Ru/C) processes.

**Table 2:** Dissociative and associative mechanisms for ammonia synthesis [23].

	Dissociative Mechanism	Associative Mechanism
(1)	N_2_ (g) + * → N_2_*	N_2_ (g) + * → N_2_*
(2)	N_2_* + * → 2N*	H_2_ (g) + * → H_2_*
(3)	N* + H* → NH* + *	H_2_* → 2H*
(4)	NH* + H* → NH_2_* + *	N_2_* + 2H* → N_2_H*
(5)	NH_2_* + H* → NH_3_* + *	N_2_H* + H* → N_2_H_2_*
(6)	NH_3_* → NH_3_ (g) + *	N_2_H_2_* + H* → N_2_H_3_*
(7)	H_2_ (g) + * → H_2_*	N_2_H_3_* + H* → N_2_H_4_*
(8)	H_2_* → 2H*	N_2_H_4_* → 2NH_2_*
(9)		NH_2_* + H* → NH_3_*
(10)		NH_3_* → NH_3_ (g) + *

## 3. Discussion

Considering the background on the TM–support interactions and, particularly, the electron transfer issue, the search for suitable supports with enhanced electron donation capacity seems to be a reasonable strategy for the design of new 3rd-generation catalysts for ammonia synthesis. As explained in the previous sections, several works report a high interest to carefully consider the electronic promotion obtained when surface electrides are used as supports, given their capacity to break the scaling relation for the N_2_ dissociation, which is as limit for the kinetic performance of 2nd-generation catalysts, opening a window for exploring different new formulas [20].

Some works presented in this review support their experimental investigations with simulations, mainly based on DFT, which reinforce the improvements of 3rd-generation catalysts in terms of enhanced kinetic mechanisms. For instance, the experimental data obtained for Ru/Ca_2_NH catalysts was used to obtain microkinetic information regarding the reaction processes and mechanisms of ammonia synthesis, by means of DFT modelling [77]. The conclusions of this study led to understanding how nitrogen dissociation is promoted by the electron promotion of the TM from the support, as well as to determining the role of the hydrogen lattice (hydrogen vacancies) as a second active center for hydrogen dissociation. Interestingly, the RDS was identified as the NH_3_ formation step, with a close match between the experimental results and the DFT simulation.

According to the background state of the art, a simple analysis of the work functions of supports could be a good criterion for the selection and design of new materials, as this property has been pointed out by Hosono and coworkers as a crucial parameter for electride materials [79].

However, as analyzed in Section 2.4., there are many other aspects with a strong influence on the performance of 3rd-generation catalysts for ammonia synthesis in addition to the electronic promotion in terms of the work function gap between TM and support, such as the surface properties of the catalyst and the TM dispersion on the support, the materials and methods for the synthesis of the catalyst, the operating reaction conditions or the addition of secondary species into the support, the tuning of internal structures or structural configurations in intermetallic materials, thus the work function gap between TM and support cannot solely explain differences between materials. Nevertheless, most supports that ensure the behavior of surface electride to catalysts have work functions in the range 2.1–2.4 eV [79]. Therefore, the use of low work function supports seems to be a reasonable first approach to screen new materials.

**Figure 7:**
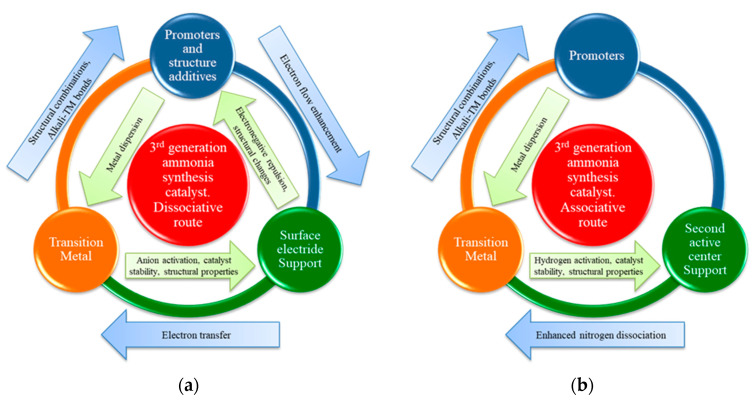
Conceptual approach for the requirements of new 3rd-generation catalysts for the (**a**) dissociative route and (**b**) associative route processes.

To summarize all the ideas presented in this work, a general scheme about the action of the 3rd-generation catalysts is shown in the Figure 7, which can be used as a conceptual approach for the requirements of new materials. An approach for general dissociative mechanism-like materials is presented in Figure 7a, while a scheme for catalysts that follow the associative route is shown in Figure 7b.

## 4. Conclusions

The present study highlights the fundamentals of thermocatalytic ammonia synthesis in terms of the reaction mechanisms, as well as the historical evolution of the 1st- and 2nd-generation ammonia synthesis catalysts, with a particular emphasis on the metal–support interactions for 3rd-generation catalysts.

For the electride-like supports, N_2_ cleavage is no longer the RDS, but there is a switch towards NH_x_ species formation and continuous free electrons migration prevents hydrogen poisoning.

Surface electrides, particularly hydrides and amines, are promising supports under mild conditions to date, since the deposition of TMs on them ensures a continuous supply of both electrons and H^−^, with activation energies in the range 20–66 kJ·mol^−1^. Furthermore, the addition of secondary species such as electronegative and basic elements seems to provide surface electrides a superior performance. In addition, internal structure tuning of novel materials and the coupling between structure versatility and stability of intermetallic compounds are promising approaches for high performance materials design.

However, many other factors influence ammonia synthesis, like TM precursor forms, the deposition process, TM loading, catalyst structural properties and operating conditions, thus a direct correlation between the electron donation of materials described by the simple form of the work functions and their performance is not truly representative, although it leads to an initial approach for the search for more efficient ammonia synthesis catalysts.

Finally, although there have been significant advances in recent decades in terms of ammonia synthesis performance and kinetic mechanisms for the new catalysts developed, there are several constraints to be solved in order to achieve technological implementation. For instance, the design of non-noble and stable materials, since some of them, such as hydrides and nitrides, are not stable under ambient conditions and handling under inert atmospheres is required. The search for a reasonable formula to couple ammonia synthesis with renewables, avoiding transient influences, should also be addressed. In addition, simple and scalable synthesis procedures are still necessary, since most novel catalysts are produced at a small scale following complex methodologies, which can hinder the industrial production of these materials.

## Data Availability

No new data were created.
